# The Truth and Falsehood of the So-Called Cold Water Cure

**Published:** 1841-07

**Authors:** 


### Art. XIV.

*Wahres und Falsches in der sogenannten Wasserheilkunde.* Von Dr. H. Claessen.—*Köln*, 1840. 12mo, pp. 127.

*The Truth and Falsehood of the so-called Cold Water Cure.* By Dr. H. Claessen.—*Cologne*, 1840.

The medicine of all barbarous nations is simple; a few well-known herbs, a few animal preparations make up mostly their whole materia medica. Superstitious observances fill up the place of medical treatment, and in the cure of almost all diseases, fire and water, whose effects are so striking as to impress the most ignorant, play a prominent part. Our own ancestors and the northern nations in general ascribed peculiar virtues to water. Standing on the river's banks they worshipped, they divined with the smooth stones of the stream, and maidens often sat all night at a spring, *waking the well.* Petrarch tells us how, in accordance with an old custom, the banks of the Rhine at Cologne were covered on St. John's eve at sunset, "præclaro et ingenti mulierum agmine,' who with many ceremonies and muttering a charm bathed their arms and hands in the stream. Water dipped from a running stream at twelve o'clock on Christmas night was reputed a cure for colic; that obtained at midnight of St. John's day was deemed a febrifuge. Many non-medicinal springs had a high reputation; some restored the old to youth; and tales were not wanting of how persons cut in pieces, like Æson of old, had emerged whole from their waters. They were visited from afar, and people sojourned near them for the sake of bathing in them and drinking their waters.

Of these wonder-working springs no other memorials now remain but names, such as Holywell in England, Heilbrunn, Heiligenbrunn, &c. in Germany, except in some secluded spots where old customs and old superstitions long linger. In such a place it was (at Gräfenberg, a little village in Silesia) that Vincent Priessnitz, rather more than ten years ago, first employed on animals that practice which has since become so generally adopted in Germany. The fame of his cures spread, and we doubt not that many an old tradition recovered strength as the simple people flocked from the neighbourhood to be healed by the Silesian peasant. Who would then have anticipated that within so short a time the Gräfenberg water-doctor should be talked of from one end of Germany to the other; that more than twenty different institutions should be formed for carrying out his practice; and that his empiricism should be styled a system, and, dignified with a Greek name, afford, under the title of Hydropathy and Hydriatics, materials for a hundred publications on its merits and defects? Such, however, has been the case; and if it has been found possible to induce several hundreds of persons annually to submit to a diet homely and often coarse, to drink some quarts of cold water, and to be dipped or pumped upon more than once daily for weeks together, or to undergo a process which causes most profuse perspiration and makes the skin break out in boils, it is surely worth while to enquire, with Dr. Claessen, what may be the measure of truth or falsehood in a method so little in accordance with our ordinary therapeutic proceedings.

In this publication the author has had two objects: the one, to "check the unbounded exaggeration of the advocates of hydriatrics, the other to obtain a scientific ground from which to enter upon a practical investigation of this new mode of treating disease." The latter is, however, by no means an easy task, for the whole literature of the subject does not, as Dr. Claessen assures us, contain a single trustworthy and carefully recorded case. Many debatable points therefore are left untouched, and the author contents himself with describing the proceedings of the hydropathists, and explaining their alleged effects in accordance with the common principles of science. Following Dr. Claessen as our guide, we shall endeavour as briefly as possible to explain to our readers the practice, and, as far as we can collect them, the principles of hydropathy.

Priessnitz, the originator of the cold-water cure, does not seem to be by any means chargeable with all the follies and extravagancies which have been perpetrated by his followers. The pretension that all diseases are curable by cold water appears indeed to have been in a great measure the result of a government ordinance which, while it permitted him to use water in the treatment of diseases in any way which he chose, absolutely interdicted the employment of any other remedy whatever. His ingenuity thus put to task, he devised various modes of applying his one means; and, being a man of considerable tact and acuteness, met with great success in his practice. A kind of system of treatment was thus formed, from which all remedies but one were of necessity excluded, and the next step was to represent cold water as a universal panacea, a sort of *elixir vitæ,* and all other means of cure not only as unnecessary, but as being actually hurtful. Too ignorant to explain the reasons which guided him in his practice, Priessnitz abandoned that task to his followers, men who differed little from their master, save that to ignorance they added impudence. At first there was a debate among them whether the principles of allopathy or of homœopathy were those to be adopted in expounding their practice; at length they hit upon a solution of their difficulty in the discovery that both were erroneous, and that the only true medical doctrine was that of hydropathy. As far as we can gather from the account of Dr. Claessen, hydropathy appears to be a sort of eclectic system, or compound of Brunonianism and of that humoral pathology which for centuries has been current among the vulgar.

We are not aware whether any work has been published setting forth the principles of hydropathy, a creed of the water-doctors, but we suspect not, for Dr. Claessen does not allude to any such. We pass over his own remarks upon the action of cold, which, though sensible, are not new, and copy from p. 25 of his book the following scheme of the different modes of employing cold water in an institution for the cold-water cure:

"A. External employment of cold water.
I. General irritation of the skin by cold water.
1. Simple irritation by cold water.
1. *a.* Washing.
2. *b.* Plunging.
3. *c.* Affusion.
4. *d.* Cold baths.
5. *e.* Cold baths after previous sweating.

2. Irritation by cold water, accompanied with mechanical action on the skin.
6. *a.* Cold washing, with brushing the skin.
7. *b.* Shower baths.
8. *c.* Douches.

II. Partial irritation of the skin by cold water.
1. Simple irritation by cold water.
9. *a.* Application of wet compresses.
10. *b.* Partial baths, hip and foot baths.

2. Irritation by cold water, accompanied with mechanical action on the skin.
11. *a.* Water falling in drops upon a part.
12. *b.* Water propelled in a slender stream against a part.
13. *c.* Douches.

B. Internal employment of cold water.
14. *a.* Drinking.
15 *b.* Clysters.
16. *c.* Injections of cold water into the different cavities of the body."

The first four methods of applying cold water require no detailed explanation. The fifth is the great remedy of the water-doctors, though by no means peculiar to them, but a modification of that kind of bath which is used, not only by the Russians, and other nations of Northern Europe, but also by the North American Indians. "They have a cure for some diseases," quaintly says good old Cotton Mather of the Indians, "even a little cave; after they have terribly heated it, a crew of them go and sit there with the priest, looking in the heat and smoke like so many fiends, and then they rush forth on a sudden and plunge into the water: how they escape death instead of getting cured is marvellous." The process in a hydropathic institution, as described by Mr. Lee in Part II. of his useful little work on the Baths of Germany, p. 121, is as follows:*

"Each patient is awakened about five o'clock in the morning by an attendant, by whom the process of *emaillottage,* or wrapping up, is performed. A blanket or woollen covering is first bound firmly round, so as to envelope the whole body, the face only being left exposed; over this are placed one or two feather beds, or eyderdown coverings, and over these again a second blanket is bound round the body, which, thus enveloped, appears to be about twice its natural size. After a time copious perspiration is induced, the window of the room is then thrown open to admit fresh air, and cold water is given to the patient at short intervals to promote the perspiration, and prevent his being weakened by its quantity, which in some instances is so profuse as to soak through all the coverings, and the mattrass of the bed. When the perspiration has continued for the period that is deemed advisable (sometimes as much as two hours) the coverings are all removed except the first blanket; a cloak being thrown over the patient, and slippers placed upon his feet, he descends quickly to the bath; and first dipping his hands and face for a second or two, throws off the blanket and plunges into the water at a temperature varying from nine to twelve degrees of Reaumur, while the perspiration is still streaming from the surface of his body; the duration of the bath is only a few seconds in some instances, in others it extends to five minutes, or even a longer period, brisk motion and friction of the surface being enjoined."

In those cases where the simple *emaillottage* does not produce per-

* The Baths of Central and Southern Germany. By Edwin Lee, Esq. London and Paris, 1841. 12mo, pp. 134.

spiration, Priessnitz wraps the patient up in a sheet wrung out of cold water, which Dr. Claessen (p. 29) asserts to be a never-failing means of exciting the action of the skin. The degree of reaction to be excited is carefully regulated by at first merely sponging the patient with cold water, and gradually proceeding to plunging, effusion, and at length to the use of the cold bath. Our author states that reaction invariably follows the application of the water, consequently that there is no danger of catching cold, but on the contrary the sensation produced by this treatment is most delightful. One superiority of Priessnitz's plan over that adopted in the ordinary Russian baths, is that the patient constantly respires cool air instead of breathing in an intensely hot atmosphere, which necessarily interferes with respiration and occasions great congestion about the head and chest.

Local applications of cold water are likewise extensively employed by the hydropathists, as in the case of wet compresses which are dipped in cold water and worn during the whole day over the seat of any organ, as the liver, heart, or stomach, which may be supposed to be especially affected. It is asserted that patients soon become reconciled to these applications. The other modes of employing cold water locally which are given in the scheme, seem to call for no explanation.

Such is the armamentarium of the Gräfenberg water-doctor and his followers, with which they profess to cure all diseases. That powerful effects are produced by some of their modes of treatment is undeniable, and the testimony of all persons concurs in representing the flow of perspiration produced by a single employment of the cold bath after sweating as enormous, while its use is often persevered in for months. Dr. Claessen tells us that the perspiration under these circumstances often assumes a very peculiar odour which is regarded by the water-doctors as an infallible sign of the elimination of the morbific virus. A very apocryphal tale is related by Mr. Lee, on the authority of one of these gentlemen, of a lady "who had taken sulphur-baths seven years previously, but who had not used sulphur since that period. After she had been pursuing the cold-water cure for some time, her room smelt of sulphur, a bracelet she wore became tarnished, and her linen was stained of a yellowish colour." Much, however, as a stinking perspiration is to be desired, a patient is to consider himself doubly fortunate if his skin should become covered with eruptions, and especially if boils in great number should break out on his person.

"Hydropathy," says Dr. Claessen, "attaches peculiar value to the purulent contents of these boils which are supposed to be made up of the diseased matter. . . . . This unusual commotion of the different fluids does not take place without a deep impression being made on the organism. Congestion of various organs occurs, severe pain is experienced in different parts more especially in such as are diseased, the activity of one or more organs of the senses is stimulated to a morbid and even to a painful degree, with all of which occurrences hydriatrics are familiar under the name of excitement, (aufregung.) In the further course of the treatment, and during the methodical increase of its activity, a regular fever is induced, which according to the statements of the water-doctors is usually followed by the cure of the disease, and to which they have given the name of crisis. It is, they assert, only in exceptional cases that the first appearance of the fever does not prove critical, but even in those instances its more energetic return and the consequent cure of the disease may be confidently anticipated. It is not unusual for the crisis to be ushered in by stormy and even dangerous symptoms, for the relief of which hydropathy, in accordance with its exclusive tendencies, employs no other remedy than water. After the fortunate termination of the crisis, the patient is looked upon as cured, but it is considered desirable that the derivation of the fluids towards the surface should be gradually diminished, which is done by the employment of the so-called after-cure." (pp. 38-9.)

The internal employment of cold water is, as has been already mentioned, merely subsidiary to its external use, and the copious draughts of that fluid which the patients are directed to take are said to favour the outbreak of perspiration as well as greatly to increase the urinary secretion. Six quarts of water are regarded as a quantity which any one may boldly venture to drink every day, and at the *table d'hôte* water is the only beverage allowed. Not only are wine and all highly-seasoned meats absolutely interdicted, but one of the dogmas of hydropathy is that all food ought to be taken cold rather than hot. For this injunction, one of the water-doctors, appealing, we presume, to the experience of his former days and humbler occupations, assigns the following reason:

"The intestines of pigs who have been fed upon hot mashes are found to be extremely readily torn, and so rotten as to be unfit for making sausages. . . . . It is therefore evident that hot food is improper since it tends to relax and soften the intestines, and that persons should partake of no food but such as is cold, or at least lukewarm." (p. 79.)

Such is a specimen of the practical reasoning of one of the apostles of the water cure. In illustration of their theories we have space only for the following explanation of the action of cold water:

"The curative power of water consists in its decomposing, purifying action. The healing effects of water result from the animosity to all organic life, with which it attempts to destroy it by reducing it to its original atoms." (p. 10.)

Enough, we trust, has already been said to convince our readers that the materials for a correct appreciation of the curative effects of cold water are not to be sought at the hands of hydropathists, whose system we can regard only as a new chapter in the long history of imposture and credulity. To Priessnitz, however, must be allowed the credit of considerable mechanical ingenuity, and of readiness in devising different methods of employing his remedy, from which some useful hints might probably be gleaned and adopted in our hospitals. The modification of the Russian bath which he has hit upon appears to have some advantages, and is undoubtedly a most energetic remedy, though we do not feel altogether that faith in its freedom from danger which is expressed by Dr. Claessen. If the advocates of the water cure should ever publish cases of which they detail the symptoms minutely and carefully, and not content themselves, as at present they do, with the assertion that they have cured so many cases of pneumonia, so many of fever, &c., we may at some future time return to the subject of hydropathy. Should they not do this, the grain of truth will nevertheless be speedily sifted from the bushel of falsehood, while hydropathy will soon be superseded by some new system of, if possible, greater pretensions and greater absurdity.

